# Identification of key genes and therapeutic drugs for cocaine addiction using integrated bioinformatics analysis

**DOI:** 10.3389/fnins.2023.1201897

**Published:** 2023-07-04

**Authors:** Xu Wang, Shibin Sun, Hongwei Chen, Bei Yun, Zihan Zhang, Xiaoxi Wang, Yifan Wu, Junjie Lv, Yuehan He, Wan Li, Lina Chen

**Affiliations:** College of Bioinformatics Science and Technology, Harbin Medical University, Harbin, Heilongjiang, China

**Keywords:** cocaine addiction, key genes, PPI network, biomarker, centrality algorithm

## Abstract

**Introduction:**

Cocaine is a highly addictive drug that is abused due to its excitatory effect on the central nervous system. It is critical to reveal the mechanisms of cocaine addiction and identify key genes that play an important role in addiction.

**Methods:**

In this study, we proposed a centrality algorithm integration strategy to identify key genes in a protein–protein interaction (PPI) network constructed by deferential genes from cocaine addiction-related datasets. In order to investigate potential therapeutic drugs for cocaine addiction, a network of targeted relationships between nervous system drugs and key genes was established.

**Results:**

Four key genes (JUN, FOS, EGR1, and IL6) were identified and well validated using CTD database correlation analysis, text mining, independent dataset analysis, and enrichment analysis methods, and they might serve as biomarkers of cocaine addiction. A total of seventeen drugs have been identified from the network of targeted relationships between nervous system drugs and key genes, of which five (disulfiram, cannabidiol, dextroamphetamine, diazepam, and melatonin) have been shown in the literature to play a role in the treatment of cocaine addiction.

**Discussion:**

This study identified key genes and potential therapeutic drugs for cocaine addiction, which provided new ideas for the research of the mechanism of cocaine addiction.

## Introduction

Drug addiction is a chronic, recurrent disorder caused by the long-term effects of drugs on the brain (Leshner, 1997). Since 1985, cocaine, a highly addictive drug that has been abused due to its excitatory effects on the central nervous system, has become one of the world's leading drugs, mostly in the Americas and Europe. According to the 2020 National Substance Use and Health Survey report released by the Substance Abuse and Mental Health Services Administration (SAMHSA), 1.9% of people 12 years of age or older in 2020 reported cocaine use in the past 12 months (NIDA, 2022). Cocaine abuse remains a major worldwide health problem (Richards and Le, 2022).

Numerous studies have shown that cocaine causes irreversible structural changes in organs such as the brain and heart (Riezzo et al., 2012; Dang et al., 2022). Research by Goertz et al. (2015) found that cocaine increases dopaminergic neurons and motor activity through midbrain α1 adrenergic signaling. It is well known that the ventral tegmental area (VTA) is an area of the midbrain. In previous studies, the VTA was found to be associated with the addictive properties of many drugs, including cocaine (Cameron and Williams, 1994). Cocaine abuse results in significant adaptation of dopamine (DA) neurons in the VTA of the midbrain (Wolf et al., 2004; Stuber et al., 2010; Mameli and Lüscher, 2011). Therefore, studies based on the midbrain region could reveal the mechanisms of cocaine addiction.

A differential gene expression analysis is commonly used for the analysis of transcriptomic datasets to explore the underlying molecular mechanisms (Liu et al., 2021). The construction of the differential gene interaction network according to differential genes has become the main method for data analysis from the system level. Generally, centrality algorithms are mainly used to identify the role of specific nodes in a network and their impact on the network, and nodes with a high centrality ranking may affect other nodes and play an important role in the network. Using a variety of centrality algorithms to analyze the network, screening the most important key genes has become the main analysis method (Chaudhary et al., 2019; Ma et al., 2021; Bhattacharyya et al., 2022; Luan et al., 2022). In the study of Zhang et al. (2020), ten different centrality algorithms in cytoHubba were used to identify key genes in the protein–protein interaction (PPI) network, and it was finally verified that the key genes were potential biomarkers or therapeutic targets for opioid addiction. In Poisel et al. (2023)'s computational biology analysis of human postmortem brain tissues with cocaine addiction, a gene ontology (GO) enrichment analysis was carried out for addiction-related CpG sites. A PPI network analysis revealed several addiction-related genes as highly connected nodes, including CACNA1C, NR3C1, and JUN. Therefore, by identifying key genes in the network, the mechanisms of the addiction process were explored in depth at the system level to explain addiction.

To date, there are no FDA-approved drug treatments for cocaine addiction (Feng et al., 2022; Shang et al., 2023), so it is necessary to explore drugs to reduce the incidence and severity of cocaine abuse. In this study, we analyzed datasets related to cocaine addiction, constructed a cocaine addiction-related PPI network to identify potential biomarkers of cocaine addiction, and finally explored potential therapeutic drugs. This could provide new ideas for studying the mechanisms of cocaine addiction and potential cocaine addiction therapeutic drugs.

## Materials and methods

**Figure 1:**
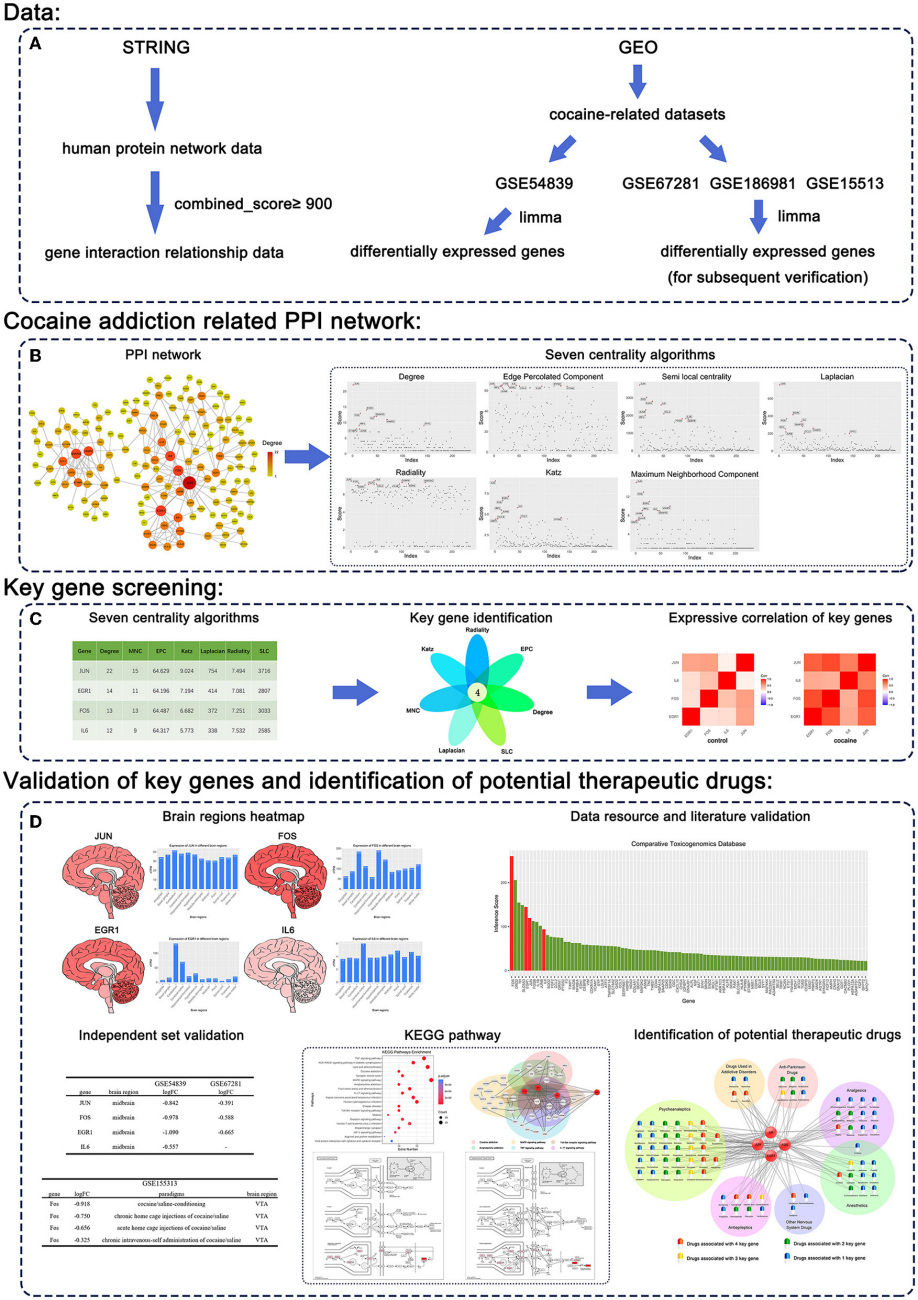
Workflow of our methodology. **(A)** Data. **(B)** Cocaine addiction-related PPI network. **(C)** Key gene screening. **(D)** Validation of key genes and identification of potential therapeutic drugs.

The procedure of our study is shown in Figure 1, and the details are described in the following sections.

### Data

Cocaine addiction-related data GSE54839 (*Homo sapiens*), GSE67281 (*Homo sapiens*), GSE186981 (*Mus musculus*), and GSE155313 (*Mus musculus*) were downloaded from the Gene Expression Omnibus (GEO) database (http://www.ncbi.nlm.nih.gov/geo). The dataset GSE54839 was chosen as our experimental set, which is based on the GPL6947 platform (Illumina HumanHT-12 V3.0 expression beadchip). This microarray-based study determined the profiles of midbrain gene expression in chronic cocaine abusers (*n* = 10) and well-matched drug-free control subjects (*n* = 10). Array-related procedures were performed in triplicate for each subject.

GSE67281 (*Homo sapiens*), GSE186981 (*Mus musculus*), and GSE155313 (*Mus musculus*) were chosen as our validation sets. GSE67281 is an expression profile in postmortem human midbrain specimens from chronic cocaine abusers (*n* = 11) and well-matched control subjects (*n* = 11). The GSE186981 is RNA-Seq data in hybrid mouse diversity panel (HMDP) mouse strains of nucleus accumbens (NAc) and prefrontal cortex brain regions (PFC). GSE155313 is the RNA-Seq data from the VTA region of mouse that underwent one of four commonly used paradigms: acute home cage injections of cocaine, chronic home cage injections of cocaine, cocaine-conditioning, or intravenous-self administration of cocaine.

Human PPI data were downloaded from the STRING database (https://string-db.org/) (Szklarczyk et al., 2021). With a combined score of >900 as the threshold, a total of 230,524 interactions between 11,763 genes were obtained.

### Cocaine addiction-related PPI network

The dataset GSE54839 was differentially analyzed using the R package “limma” to obtain their differential genes. The *p*-value of <0.05 and |log_2_F>p20mm| > 0.2630344 (i.e., fold change ≥ 1.2 or fold change ≤ 0.8) were considered statistically significant.

To obtain the interaction relationships between DEGs, the downloaded PPI data were filtered using the DEGs obtained from the microarray data GSE54839. By using the gene interactions as edges and DEGs as nodes, a differential gene network was constructed. After removing scatters from the network, the core network was defined as a PPI network related to cocaine addiction.

### Key gene identification

We proposed a centrality algorithm integration strategy to analyze genes in cocaine addiction-related PPI networks. The scores of the node in network under each centrality algorithm were calculated separately by applying a series of centrality measures, including degree, edge-percolated component (EPC), Laplacian centrality, maximum neighborhood component (MNC), Katz radiality, and semi-local centrality (SLC). The intersection of the top 10 genes of each centrality algorithm was considered the key gene.

In this study, G is the cocaine addiction-related PPI network we built, and V(G) is the collection of nodes in the network. For node x in G, N(x) is the set of direct neighbors of x in G. For collection A, |A| is used to represent the number of elements in the collection. The specific algorithms are as follows:

1. Degree (Deg)
  $$\begin{array}{l} Deg\left(x\right)=|N\left(x\right)|. \end{array}$$
2. Edge percolated component (EPC)
  $$\begin{array}{l} EPC\left(x\right)=\frac{1}{\left|V\left(G\right)\right|}\overset{1000}{\underset{k=1}{\sum }}\underset{y\in V}{\sum }\delta_{xy}^{k}. \end{array}$$
  Given a threshold of 0.5, 1,000 reduced networks were created by assigning each edge a random number between 0 and 1 and removing edges with associated random numbers less than the threshold. Let the *G*_*k*_ be the reduced network generated at the *kth* reduced process. If nodes x and y are connected in *G*_*k*_, set $\delta_{xy}^{k}$ to 1; otherwise, $\delta_{xy}^{k}=0$ (Chin et al., 2014).
3. Laplacian centrality (Qi et al., 2012):
  (1) $$\begin{array}{l} Lap\left(x\right)={Deg\left(x\right)}^{2}+Deg\left(x\right)+2\underset{y\in N\left(x\right)}{\sum }Deg\left(y\;\right). \end{array}$$
4. Maximum neighborhood component (MNC):
  (2) MNC(x)=|V(m(N(x))|,
  where *m*(*N*(*x*)) is a maximum connected component of the induced subgraph of G by *N*(*x*) (Lin et al., 2008).
5. Katz centrality:
  (3) $$\begin{array}{l} Katz\left(x\right)=\overset{\infty }{\underset{k=0}{\sum }}\overset{\left|V\left(G\right)\right|}{\underset{y=1}{\sum }}\alpha^{k}{\left(A^{k}\right)}_{xy}, \end{array}$$
  where A is the adjacency matrix of the network G with eigenvalues λ, ${\left(A^{k}\right)}_{xy}$ is the number of paths from x to y with length k, α is a damping factor and $0<\alpha <\frac{1}{\lambda_{\max}}$. In all our experiments, we chose α = 0.1 (Wei et al., 2020).
6. Radiality (rad):
  (4) $$\begin{array}{l} Rad\left(x\right)=\underset{y\in V\left(G\right)}{\sum }\frac{d+1-s\left(x,y\right)}{|V\left(G\right)|-\;1}, \end{array}$$
  where d is the diameter of the network G (Valente and Foreman, 1998).
7. Semi-local centrality (SLC):
  (5) $$\begin{array}{l} SLC\left(x\right)=\underset{y\in N\left(x\right)}{\sum }\underset{z\in N\left(y\right)}{\sum }B\left(z\right), \end{array}$$
  where B(z) is the number of direct connections and two-step neighbors for node z (Chen et al., 2012).

### Correlation analysis of key genes with cocaine addiction

The Human Protein Atlas (HPA; https://www.proteinatlas.org/) database creates a brain-centric knowledge resource on RNA and protein expression in three mammalian brains: human, pig, and mouse (Sjöstedt et al., 2020). The RNA expression of key genes in different brain regions in humans and mice was searched in the brain section of the HPA database.

The Comparative Toxicogenomics Database (CTD, https://ctdbase.org/) was used to obtain associations between key genes and cocaine addiction. In the CTD database, the inference score reflects the degree of similarity between the CTD chemical–gene–disease network and a similar scale-free random network (Davis et al., 2023). The higher the score, the higher the degree of association between the disease and the gene.

The role of key genes in the mechanism of cocaine addiction was identified by text mining in the PubMed database. The search keywords were “cocaine addiction” and the four key genes.

Three independent sets GSE67281 (human), GSE186981 (mouse), and GSE155313 (mouse) were used for pre-addiction and post-addiction differential expression analyses to verify key genes, and the threshold and differential analysis methods were consistent with those of the experimental set GSE54839.

The R package “homologene” was used to search for homologous genes between the human and the mouse. The “homologene” package is a package based on the NCBI HomoloGene (https://www.ncbi.nlm.nih.gov/homologene/) database. The HomoloGene database is a system that can automatically detect congeners in human and mouse genes (NCBI Resource Coordinators, 2014).

To investigate the possible molecular mechanisms of key genes for cocaine addiction, we used the Kyoto Encyclopedia of Genes and Genomes (KEGG; http://www.kegg.jp/ or http://www.genome.jp/kegg/) database for enrichment analysis. An adjusted *p*-value of <0.05 was considered to be statistically significant.

### Potential therapeutic drug identification

In order to identify potential therapeutic drugs for cocaine addiction, a network of targeted relationships between nervous system drugs and key genes was constructed. Nervous system drug information was retrieved from the ATC classification system of the Drugbank (www.drugbank.ca) database (Wishart et al., 2018). The targeted effects of key genes with nervous system drugs were reflected in the CTD, where drugs that affect the expression level of genes were our screening criteria. According to the targeting relationship between key genes and nervous system drugs, a targeted relationship network between nervous system drugs and key genes was constructed. Cytoscape was used to visualize this network. Finally, the network was analyzed to screen for potential therapeutic drugs for cocaine addiction.

## Results

### Cocaine addiction-related PPI network

**Figure 2:**
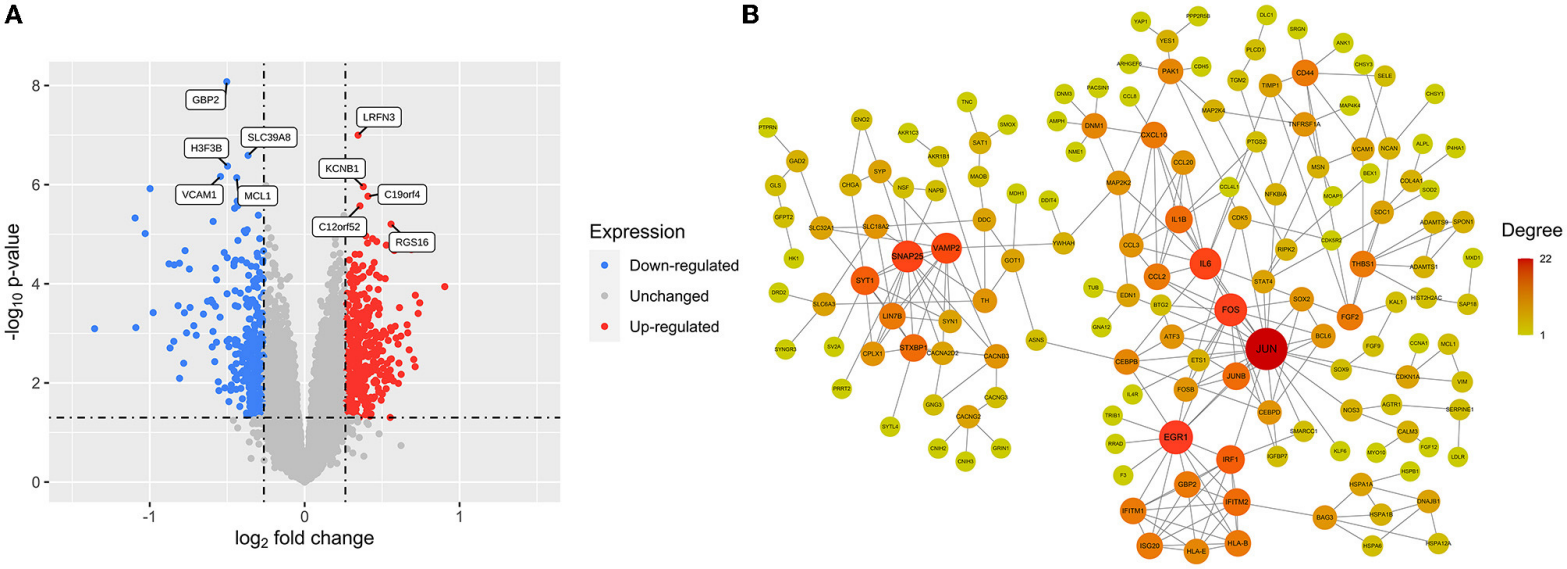
DEGs and cocaine addiction-related PPI network: **(A)** A volcano plot of 724 DEGs. Red: upregulated genes; blue: downregulated genes; gray: unchanged genes. **(B)** Cocaine addiction-related PPI network. The higher the degree value, the larger the node.

A total of 724 DEGs were identified from the GSE54839 dataset, including 409 up-regulated genes and 315 down-regulated genes (Supplementary Table 1). The volcano plot was plotted with the “ggplot2” package in R software to visualize the identified DEGs (Figure 2A).

The differential genes were mapped to the downloaded PPI data to obtain a differential gene network consisting of 236 nodes and 316 edges. Removing scatter points in the network, the core network had a total of 153 nodes and 263 edges, which was defined as a cocaine addiction-related PPI network. Cytoscape software was used to visualize the network (Figure 2B).

### Key gene

**Figure 3:**
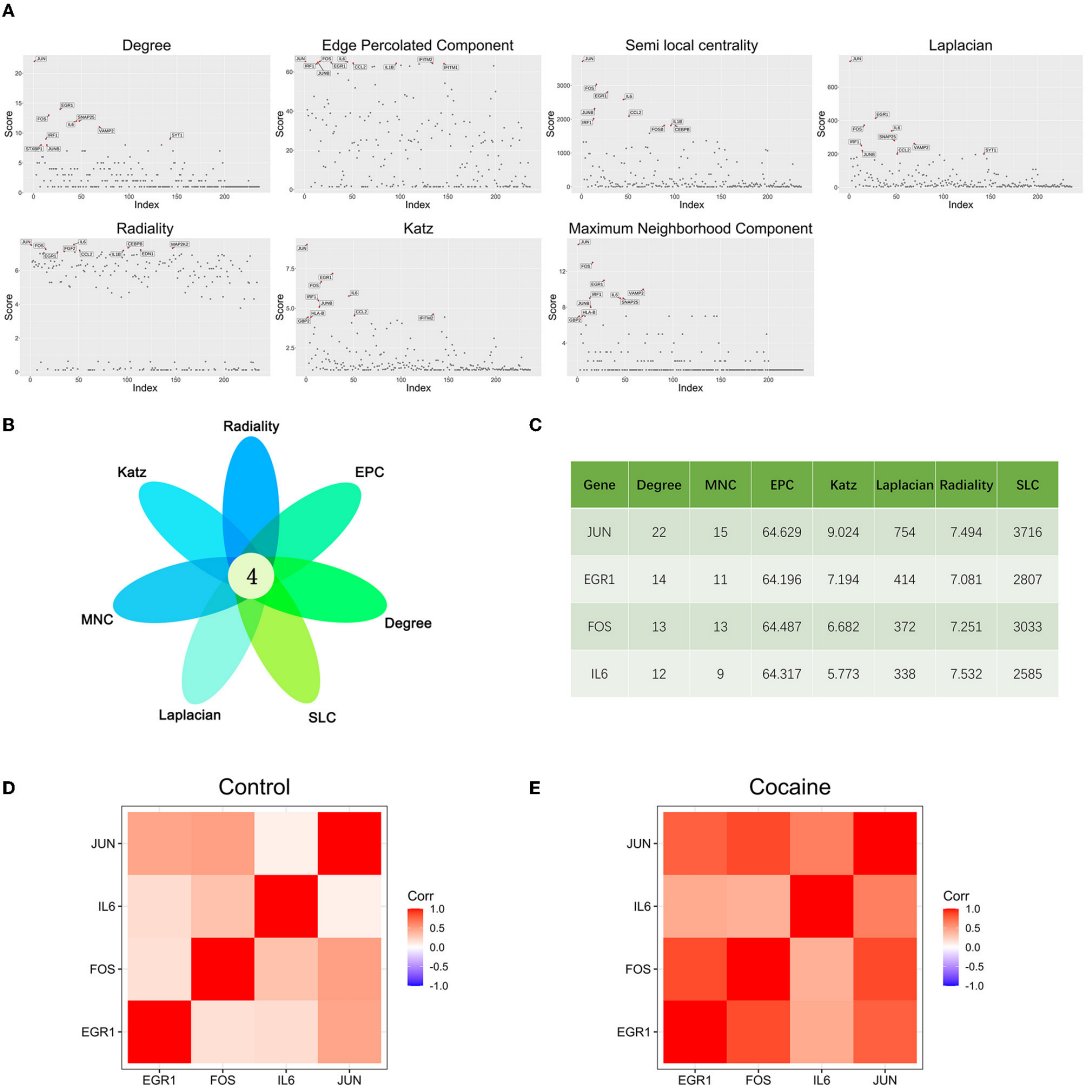
Key gene identification: **(A)** Seven centrality algorithms calculate the distribution of scores for all nodes in the PPI network, and the red signal marks the top ten genes in the score. **(B)** The top 10 hub genes in the PPI network were identified by seven centrality algorithms and overlapped to obtain four key genes. **(C**) Seven centrality algorithm results for four key genes. **(D)** Co-expression analysis heat map of four key genes in samples from drug-free control subjects and chronic cocaine abusers. **(E)** Co-expression analysis heat map of four key genes in samples from drug-free control subjects and chronic cocaine abusers.

The scores of each node in the cocaine addiction related PPI network were calculated separately using seven different centrality algorithms (Figure 3A, Supplementary Table 2). Based on our proposed centrality algorithm integration strategy, the top ten genes scored by each algorithm were selected, and their intersections (FOS, IL6, EGR1, and JUN) were regarded as key genes (Figure 3B). The scores of the seven centrality algorithms for the four key genes are shown in Figure 3C.

We performed correlation analyses for four key genes in non-drug control participants (Figure 3D) and chronic cocaine abusers (Figure 3E), respectively. The correlation heat map showed that all four key genes were positively correlated, and the correlation showed a significant increase in the cocaine group. It suggested that these four key genes might be more closely related to each other and had synergistic effects during addiction. In addition, the expression of the IL6 gene was the lowest of the four key genes.

### Expression of key genes in brain regions

**Figure 4:**
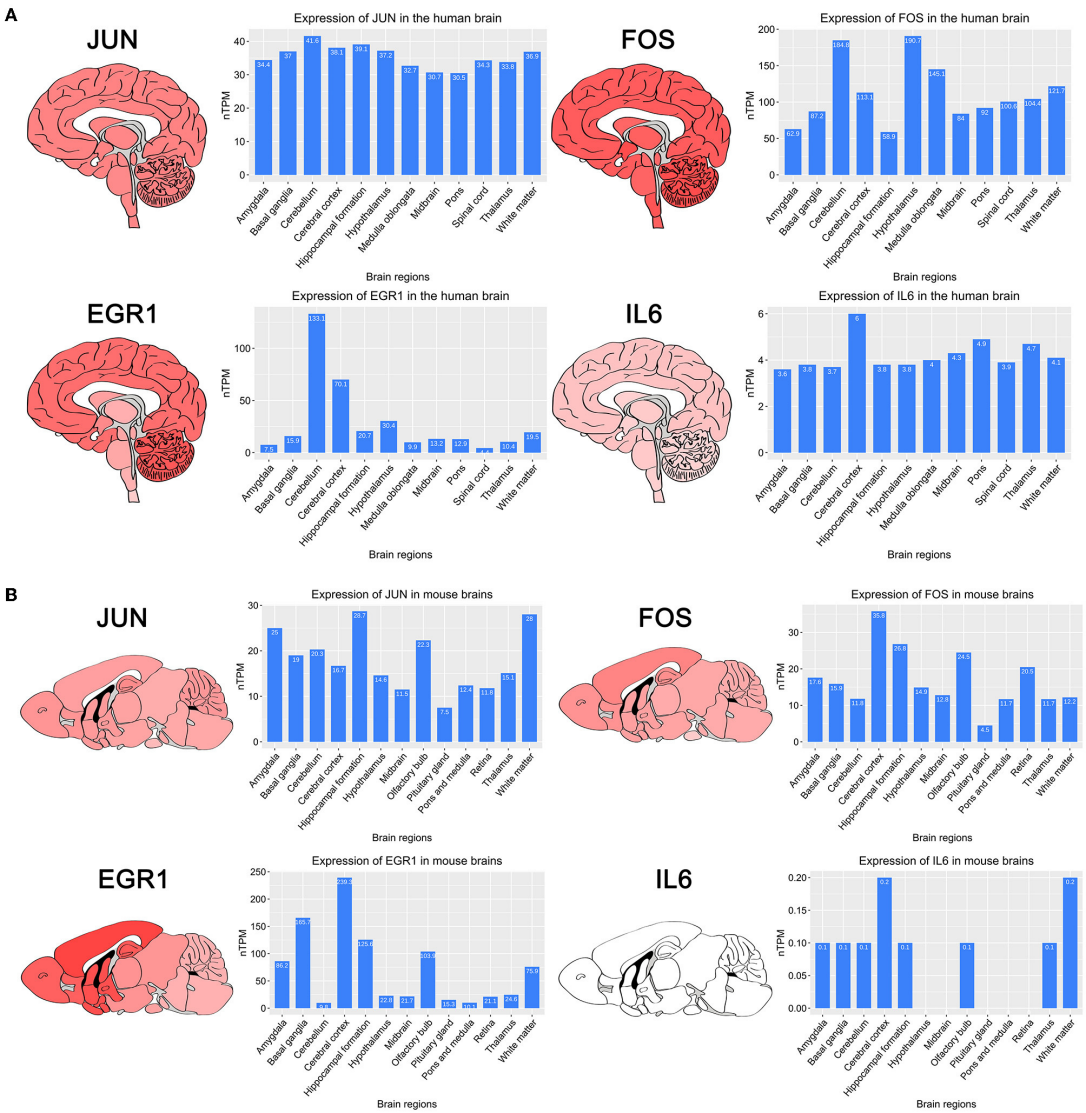
Brain regions heatmap of four key genes and histograms of expression of different brain regions obtained in the HPA database. **(A)** Expression of four key genes in human brain regions. **(B)** Expression of four key genes in mouse brain regions.

To investigate the expression of four key genes in the brain, we searched the HPA database for the expression of four key genes in different brain regions in the human and mouse (Figures 4A, B). The results showed that four key genes were expressed in all regions of the human brain, and IL6 was expressed in the human midbrain region lower than the other three genes, which is consistent with our findings. IL6 was not detected in the mouse midbrain, hypothalamus, pituitary gland, retina, pons, and medulla.

### Correlation analysis of CTD databases

**Figure 5:**
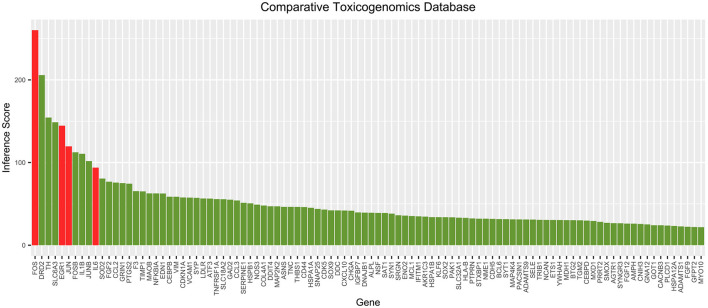
Top 100 correlation scores between all genes in the cocaine addiction-related PPI network and cocaine addiction. Red represents key genes and green represents other genes in the network.

The correlation scores between each gene in the cocaine addiction-related PPI network and cocaine addiction were searched in CTD. The scores for the top 100 genes are shown in Figure 5. It showed a higher degree of association between the four key genes and cocaine addiction. CTD showed that FOS and EGR1 could be biomarkers of cocaine addiction or play a role in addiction, and EGR1 could be a gene for a therapeutic target in the treatment of cocaine addiction.

### Literature validation

The PubMed database showed that four key genes were all associated with cocaine addiction. Both FOS (Fos proto-oncogene) and JUN (Jun proto-oncogene) are members of the AP-1 transcription factor complex. Multiple studies have shown that cocaine affected the expression of FOS proteins (Todtenkopf et al., 2002; Imam et al., 2005; Larson et al., 2010; Lobo et al., 2010). Zhang et al. (2004) prepared CPu extracts from D1 and D3 receptor mutant mice and wild-type control littermates at different time points after cocaine injection and found that ERK activation mediates acute cocaine-induced expression of c-fos (Fos). The study by Xu (2008) found that c-fos might mediate cocaine-induced persistent changes by regulating the formation of AP-1 transcriptional complexes and gene expression. Previous studies have demonstrated that cocaine causes increased expression of the JUN protein (Malaplate-Armand et al., 2005; Paletzki et al., 2008). Cocaine affects the expression of the JUN protein (Imam et al., 2005).

There are some studies proving that EGR1 (early growth active protein 1) and c-fos expressions are reduced after cocaine induction (Helton et al., 1993; Ennulat et al., 1994). In experiments on mutant mice by Valjent et al. (2006), EGR1 was found to play a vital role in cocaine-related behavior. Humblot et al. (1998) found that acute cocaine administration was effective in inducing c-FOS and EGR-1 direct early genes, and cocaine-induced EGR-1 and c-FOS expression was significantly reduced in brain regions of rats.

IL6 (interleukin-6) is a pro-inflammatory cytokine. The study by Halpern et al. (2003) showed that men and women respond weakly to pro-inflammatory challenges to IL6 after intravenous cocaine. In experiments measuring changes in IL6 levels in crack cocaine-dependent adolescents after 21 days of withdrawal, it was found that IL6 was elevated in patients on admission compared to the control group (Pianca et al., 2017).

### Independent set analysis

**Figure 6:**
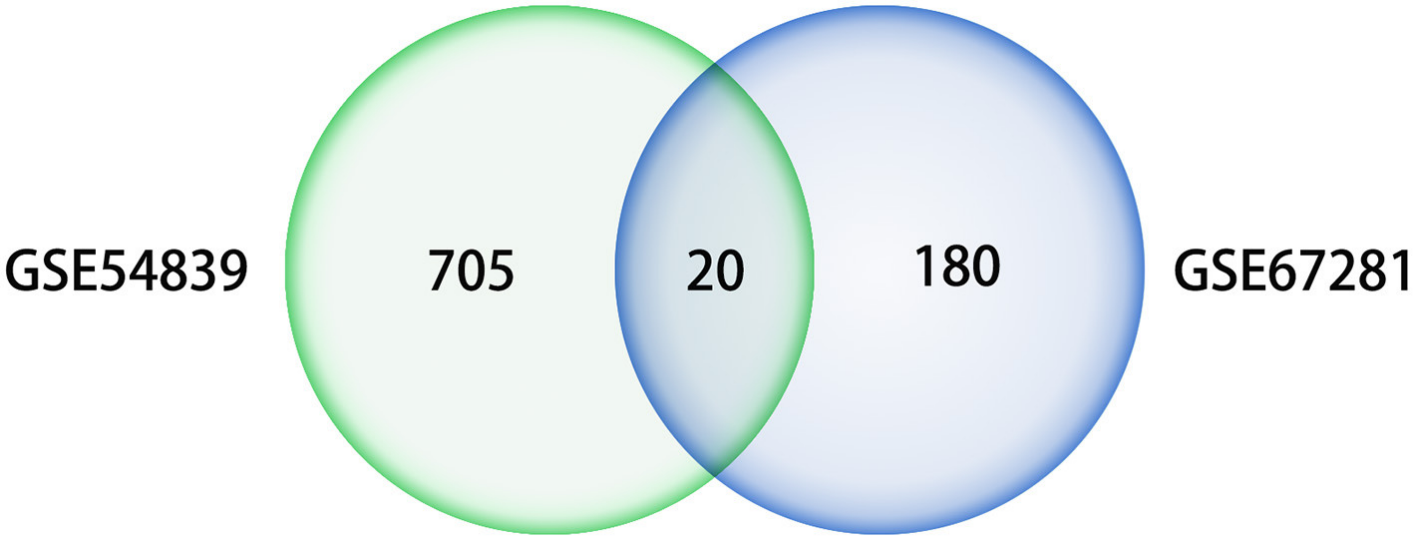
Venn diagram of differential genes in datasets GSE54839 and GSE67281.

A differential expression analysis was performed on the human dataset GSE67281, which is the expression profile of human cocaine abusers in the midbrain region. A total of 200 DEGs were identified after annotation, including 110 upregulated genes and 90 downregulated genes. There were 20 intersecting genes in the datasets GSE54839 and GSE67281 (Figure 6), including the key genes JUN, FOS, and EGR1, all of which were downregulated in the addictive state (Table 1), while the IL6 gene was not annotated in GSE67281.

**Table 1:** Differential expression of key genes in GSE54839 and GSE67281.

		**GSE54839**	**GSE67281**
**Gene**	**Brain region**	**logFC**	**logFC**
JUN	Midbrain	−0.842	−0.391
FOS	Midbrain	−0.978	−0.588
EGR1	Midbrain	−1.090	−0.665
IL6	Midbrain	−0.557	–

A differential expression analysis and a homology analysis were performed on the mouse dataset GSE155313 from the VTA brain region, and the Fos gene was identified as a downregulated differential gene under four different conditions. The degree of difference in the Fos gene was not the same between chronic and acute home cage injections of cocaine, and even greater in chronic home cage injections of cocaine condition (Table 2). It suggested that the Fos gene plays a crucial role in long-term addiction.

**Table 2:** Differential expression of key genes in GSE155313.

**GSE155313**			
**Gene**	**logFC**	**Paradigms**	**Brain region**
Fos	−0.918	Cocaine/saline-conditioning	VTA
Fos	−0.750	Chronic home cage injections of cocaine/saline	VTA
Fos	−0.656	Acute home cage injections of cocaine/saline	VTA
Fos	−0.325	Chronic intravenous-self administration of cocaine/saline	VTA

The JUN, FOS, and EGR1 genes were shown to be downregulated differential genes in the human validation set, and the FOS gene was also downregulated in the mouse validation set, which is the same as the experimental set. Analysis of the independent sets showed that the key genes we identified were well-confirmed.

### Pathway verification

**Figure 7:**
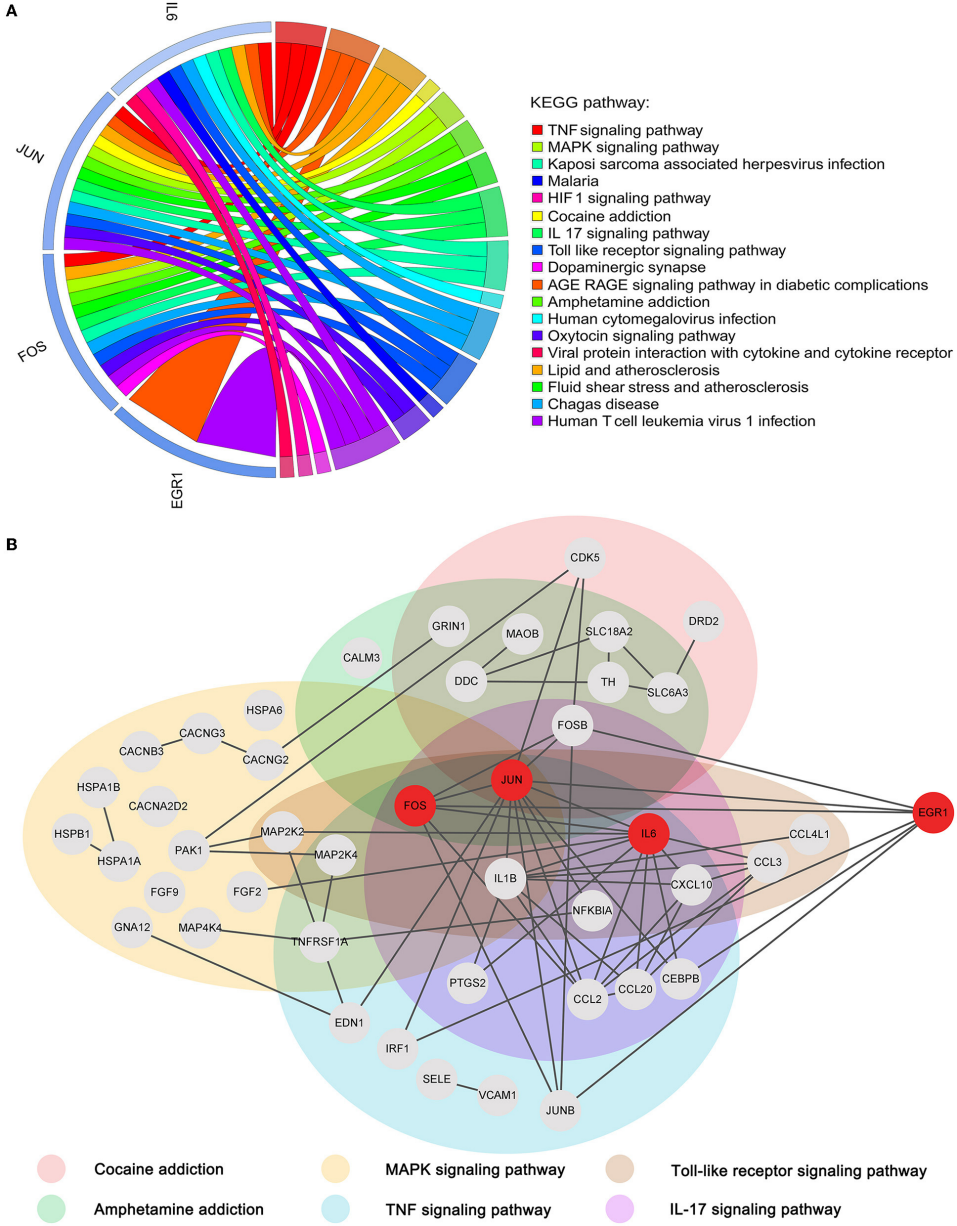
Functional enrichment analysis of key genes. **(A)** Chord diagram of four key genes vs. the top 20 KEGG pathways. **(B)** Subnetwork associated with key genes and addiction-related KEGG pathways.

To reveal the roles of the key genes, we performed a KEGG enrichment analysis of all the genes in the cocaine addiction-related PPI network. A total of 75 KEGG pathways were enriched (Figure 7A). The four key genes were mainly enriched in the TNF signaling pathway, cocaine addiction, amphetamine addiction, IL-17 signaling pathway, MAPK signaling pathway, and Toll-like receptor signaling pathway. Previous studies have shown that these pathways were all linked to cocaine addiction (Northcutt et al., 2015; Lewitus et al., 2016; Brown et al., 2018; Ganguly et al., 2019; Montesinos et al., 2020; Bingor et al., 2021). The subnetwork associated with the key genes and their enrichment pathways (Figure 7B) showed that the four key genes were closely connected in the network, among which JUN, FOS, and IL6 were enriched into multiple pathways, and EGR1 was closely related to these pathways and played a very important synergy.

**Figure 8:**
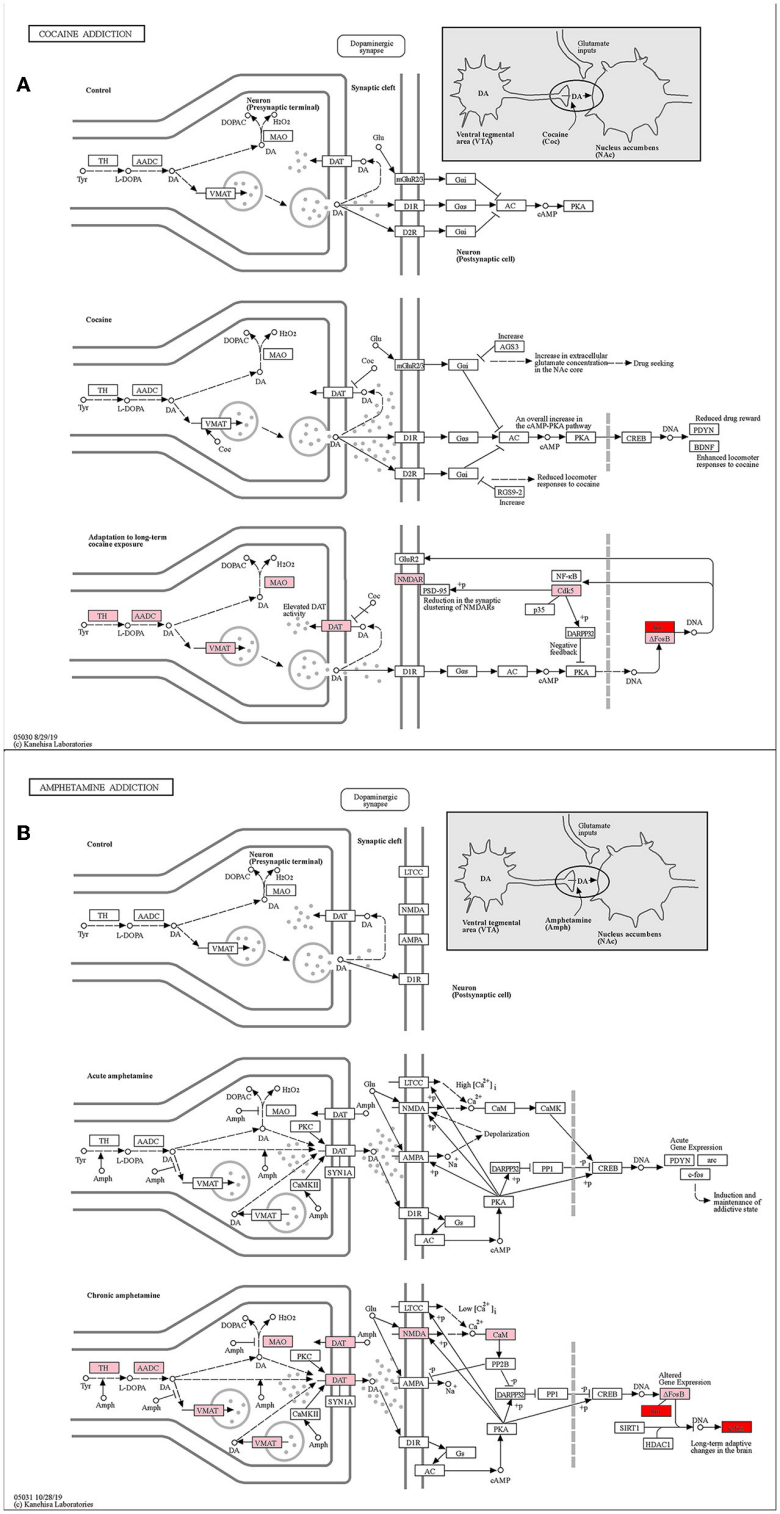
KEGG pathway map (Kanehisa and Goto, 2000; Kanehisa, 2019; Kanehisa et al., 2023). **(A)** Cocaine addiction pathway. **(B)** Amphetamine addiction pathway. Pink represents differential genes and red represents key genes.

The cocaine addiction pathway and the amphetamine addiction pathway are two enriched addiction-related pathways (Figures 8A, B). They have similar addiction mechanisms, and both have enhanced firing activity of dopamine neurons in the VTA of the midbrain, resulting in enhanced dopamine release from the NAc.

With the stimulation of addictive drugs, the FOS gene induces and maintains an addictive state in the short term of addiction. In human who achieve long-term addiction after further drug use, the FOS gene causes long-term adaptive changes in the brain, and the JUN gene dimerizing with ΔFosB leads to an increased cocaine response. ΔFosB desensitizes c-fos mRNA induction after chronic amphetamine exposure (Renthal et al., 2008). Zhang et al. (2006) have shown that FOS might mediate cocaine-induced persistent changes by regulating AP-1 transcriptional complexes and target gene expression. To sum up, both our key genes JUN and FOS played important roles in the addiction pathways.

### Identification of potential therapeutic drugs for cocaine addiction

**Figure 9:**
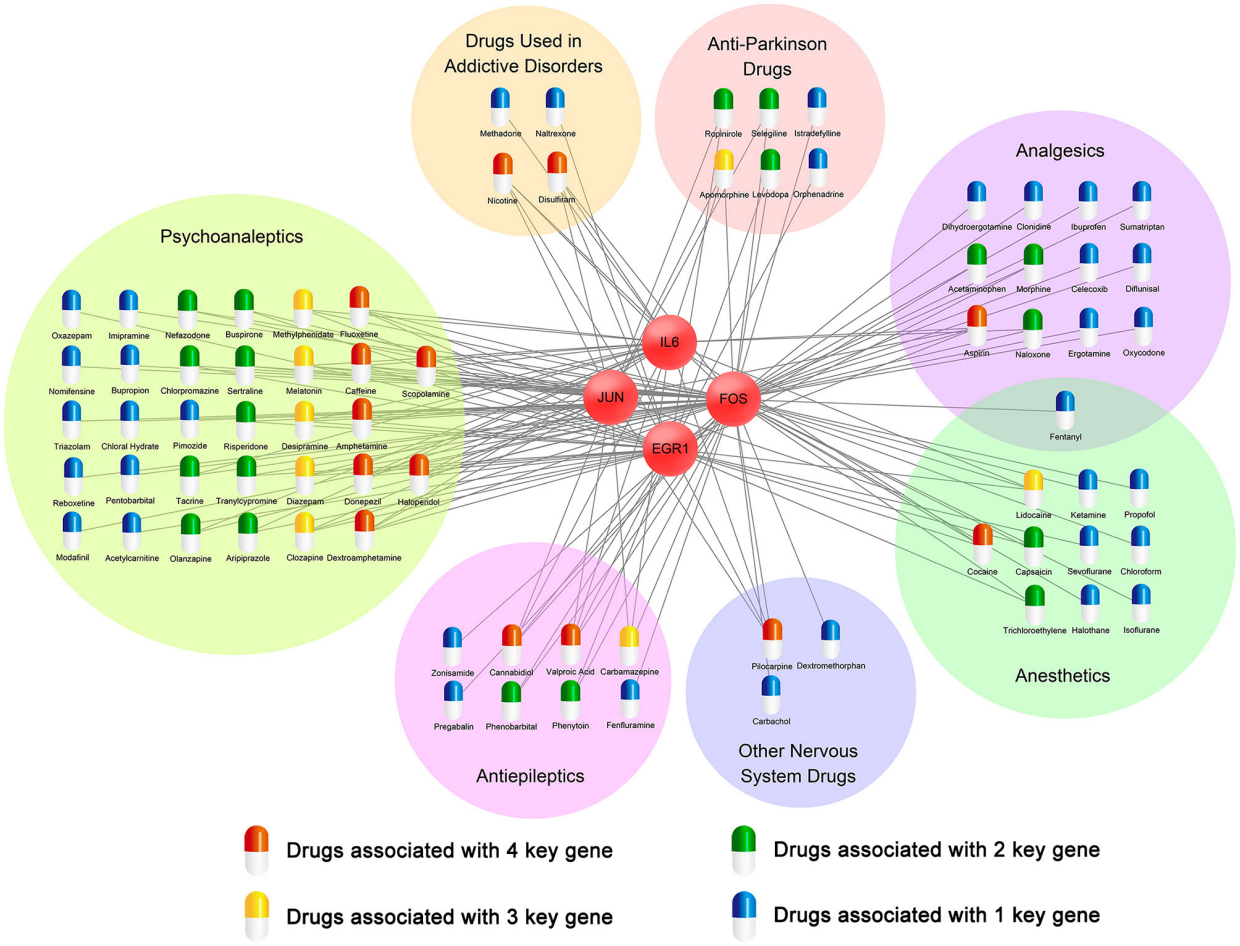
A network of targeted relationships between nervous system drugs and key genes.

To find potential therapeutic drugs for cocaine addiction, a network of targeted relationships between nervous system drugs and key genes was constructed (Figure 9). Fourteen drugs affected four genes, eight drugs affected three genes, nineteen drugs affected two genes, and thirty-five drugs affected one gene. In particular, among drugs that affected four genes, disulfiram, cannabidiol, and dextroamphetamine have been used to mitigate the cocaine response. Many studies have shown that disulfiram might reduce cocaine use in patients with cocaine dependence (Petrakis et al., 2000; Gaval-Cruz and Weinshenker, 2009; De Mulder and Dom, 2012; Kosten et al., 2013). In the experiment conducted by Petrakis et al. (2000), disulfiram inhibited dopamine β-hydroxylase, resulting in dopamine overdose and decreased norepinephrine synthesis, possibly weakening cocaine cravings, leading to reduced cocaine use. Dextroamphetamine, a central nervous system stimulant, has been found to be a treatment for cocaine dependence (Grabowski et al., 2001; Shearer et al., 2003; Palis et al., 2021; Ndiaye et al., 2022). In experiments on rats by Chiodo and Roberts (2009), sustained dextroamphetamine treatment was found to weaken the potentiating effect of cocaine. Cannabidiol (CBD) is one of the main components of cannabis, and multiple studies have shown that CBD may act as a therapeutic drug for substance abuse (Katsidoni et al., 2013; Calpe-López et al., 2019; Anooshe et al., 2021; Karimi-Haghighi et al., 2022). Recent research showed that CBD can be effective in reducing the reward and reinforcement effects of addictive drugs (Galaj et al., 2020). Among the drugs that affected the three genes, diazepam and melatonin might be useful therapeutic agents for reducing cocaine abuse (Takahashi et al., 2017; Barbosa-Méndez et al., 2021; Sanchez et al., 2022).

In twenty-two drugs affecting the expression of three or four key genes, five (disulfiram, dextroamphetamine, diazepam, cannabidiol, and melatonin) have been validated in the literature to reduce cocaine abuse and be used to treat cocaine addiction. They are distributed among drugs used in addictive diseases, psychoanaleptics, and antiepileptic drugs. We, therefore, speculated that seventeen drugs that affected the expression of three or four genes in these three classes might play the same role.

The Drugbank database was used to analyze the status of these seventeen drugs. All of them have been approved by the FDA for the treatment of other diseases, and their effects on cocaine addiction are still being studied. Twelve of these drugs, namely, disulfiram, nicotine, fluoxetine, donepezil, caffeine, amphetamine, cannabidiol, desipramine, valproic acid, dextroamphetamine, carbamazepine, and methylphenidate, are currently in clinical trials for the treatment of cocaine addiction.

The CTD was then used to analyze the relationship between seventeen drugs and cocaine addiction, and the results showed that disulfiram, dextroamphetamine, caffeine, fluoxetine, methylphenidate, desipramine, scopolamine, valproic acid, diazepam, haloperidol, donepezil, clozapine, carbamazepine, and cannabidiol were chemicals with known or potential therapeutic effects in cocaine addiction. Disulfiram, dextroamphetamine, nicotine, fluoxetine, caffeine, methylphenidate, desipramine, diazepam, scopolamine, amphetamine, haloperidol, and melatonin were chemical substances related to cocaine addiction or may play a role in the etiology of cocaine addiction. Although all seventeen drugs have been confirmed in the CTD to be associated with cocaine addiction, further research is needed to determine whether these drugs can be used to treat cocaine addiction.

## Discussion

To study the mechanisms of chronic cocaine addiction, data on chronic cocaine abuse in the human midbrain region were used for analysis. Based on the differential expression analysis, a cocaine addiction-related PPI network was constructed, and seven different network centrality algorithms were used to calculate the scores of each gene in the network separately. Finally, four key genes were screened: FOS, IL6, JUN, and EGR1. Through CTD database correlation analysis, literature verification, independent dataset analysis, and enrichment analysis, we found that the four key genes were significantly associated with addiction, and they showed more significant changes under long-term addiction. The network of targeted relationships between nervous system drugs and key genes showed that seventeen drugs targeting three or four key genes were distributed among drugs used in addictive diseases, psychoanaleptics, and antiepileptic drugs, five of which have been shown to be associated with cocaine treatment in the literature. This suggested that key genes might serve as biomarkers for cocaine addiction and that potential therapeutic drugs for cocaine addiction could be found based on key genes.

In this study, the seven centrality algorithms we used were all calculated based on the attributes of the nodes themselves. To show the importance of identifying key genes, we also used these four centrality algorithms to analyze genes in the PPI network associated with cocaine addiction. The algorithm based on the shortest path (closeness, betweenness, EcCentricity, and stress) was not considered. The results of the four unused algorithms are shown in Tables 3, 4. We obtained four key genes using seven centrality algorithms, of which three to four genes were also included in the results of these four unused algorithms.

**Table 3:** Top 10 results of three unused algorithms.

**Closeness**	**Betweenness**	**Stress**
JUN	JUN	JUN
IL6	IL6	IL6
FOS	MAP2K2	MAP2K2
EGR1	VAMP2	VAMP2
CEBPB	YWHAH	YWHAH
CCL2	FGF2	EGR1
IL1B	CEBPB	IFITM2
MAP2K2	EGR1	BAG3
JUNB	ASNS	FGF2
FGF2	GOT1	CEBPB

**Table 4:** Top 20 results of EcCentricity algorithm.

**Gene**	**EcCentricity**
GOT1	0.125
MAP2K2	0.125
YWHAH	0.125
IL6	0.125
EDN1	0.125
JUN	0.111
PAK1	0.111
TH	0.111
VAMP2	0.111
DDIT4	0.111
FOS	0.111
FGF2	0.111
DDC	0.111
TIMP1	0.111
CCL2	0.111
CCL20	0.111
CXCL10	0.111
IL1B	0.111
MDH1	0.111
CDK5	0.111

The HPA database showed that four key genes were expressed in multiple brain regions in humans and mice, so data from other brain regions in mice were used for analysis. The validation set GSE186981 was located in the NAc and PFC brain regions, and the difference analysis showed that the Fos and Egr1 genes were downregulated differential genes in both the NAc and PFC brain regions (Table 5). In the validation set of the two sets of mice, the Il6 and Jun genes were not differentially expressed genes. Human addiction to drug abuse is a long-term process, while animal model experiments are usually relatively short and may not fully mimic the process of long-term addiction in humans. In the human addiction pathway map, the FOS gene undergoes changes after acute drug administration, while the JUN gene undergoes changes after long-term addiction. The longest experimental period of the mouse validation set we used is only 7 days, which may not be enough to have formed long-term addiction, so there was no significant difference in the Jun gene. The expression of the IL6 gene was very low in both humans and mice, so we infer that it was too low to reach the difference.

**Table 5:** Differential expression of key genes in GSE186981.

**GSE186981**		
**Gene**	**logFC**	**Brain region**
Fos	−0.325	NAc
Fos	−0.318	PFC
Egr1	−0.270	NAc
Egr1	−0.316	PFC

**Figure 10:**
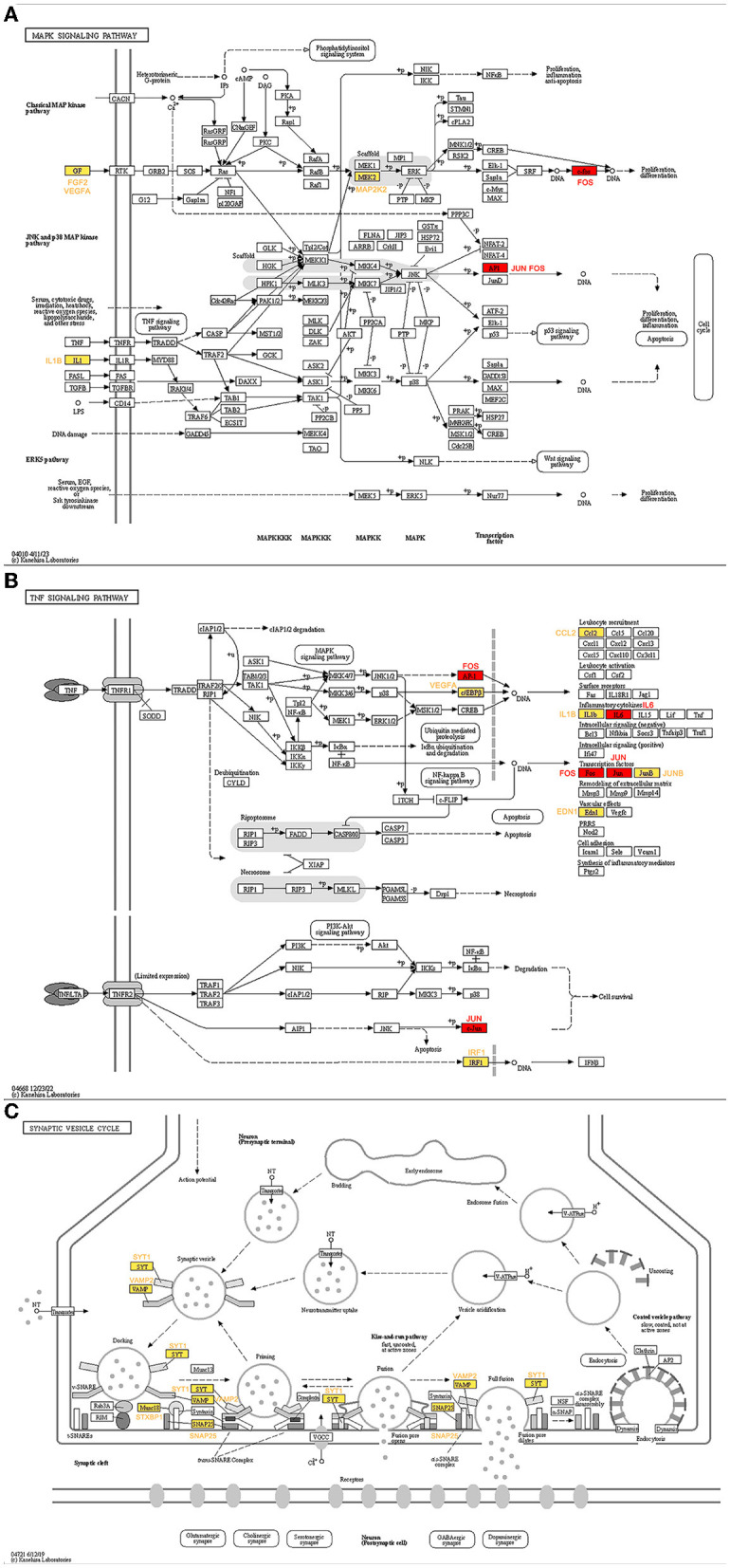
KEGG pathway map of non-key genes (Kanehisa and Goto, 2000; Kanehisa, 2019; Kanehisa et al., 2023). **(A)** MAPK signaling pathway. **(B)** TNF signaling pathway. **(C)** Synaptic vesicle cycle. Red represents key genes and yellow represents non-key genes in the top 10 of the seven centrality algorithms.

The genes ranked top in the network based on centrality algorithms were important since they were central in the cocaine addiction-related PPI network, so we analyzed the non-key genes in the top 10 genes of the seven centrality algorithms. The expression of these genes showed significant differences before and after addiction. To reveal their functions, we analyzed these genes using the KEGG database. The results showed that non-key genes were mainly enriched in the cocaine addiction pathway, amphetamine addiction pathway, MAPK signaling pathway, TNF signaling pathway, and synaptic vesicle circulation pathway, all of which were related to cocaine addiction. In the addiction pathway, long-term exposure to addictive drugs can induce a unique transcription factor, delta FosB, which can cause long-term adaptive changes in the brain. Research has confirmed that cocaine can induce the production of TNF (Kovalevich et al., 2015; Lewitus et al., 2016; Sil et al., 2019), thereby affecting non-key genes downstream of this pathway (Figure 10A). Upstream non-key genes (FGF2, VEGFA, and IL1B) affect the expression of downstream genes in this pathway (Figure 10B), while MAP2K2 regulates ERK through phosphorylation, thereby affecting cocaine addiction. Drug addiction is closely related to synapses, and non-key genes (SYT1, VAMP2, SNAP25, and STXBP1) play an important role in the synaptic vesicle circulation pathway (Figure 10C).

To date, there are too few datasets related to cocaine addiction in humans, and the sample size in our study is not very large. Therefore, the potential biomarkers and therapeutic targets of cocaine addiction identified in this study needed further experimental verification.

In summary, this study identified four key genes (FOS, IL6, EGR1, and JUN) that might be involved in cocaine addiction mechanisms and had potential roles as biomarkers and therapeutic targets for cocaine addiction. Our research provided new ideas for the study of the mechanism of cocaine addiction and was expected to help in the treatment of cocaine addiction.

## Data availability statement

Publicly available datasets were analyzed in this study. This data can be found here: https://www.ncbi.nlm.nih.gov/geo/, GSE54839, GSE67281, GSE155313, and GSE186981.

## Author contributions

WL and LC: conceptualization, project administration, and supervision. XuW, SS, HC, BY, ZZ, XiW, and YW: data curation. XuW: formal analysis and investigation. WL: funding acquisition. XuW, WL, and LC: methodology. SS, HC, BY, ZZ, XiW, YW, JL, and YH: validation. XuW and WL: visualization and writing—original draft. LC: writing—review and editing. All authors have read and agreed to the published version of the manuscript.

## Supplementary material

Supplementary Table 1 Differential expression analysis results of GSE54839.

Supplementary Table 2 Scores of seven centrality algorithms.
